# Apolipoprotein Proteomics for Residual Lipid-Related Risk in Coronary Heart Disease

**DOI:** 10.1161/CIRCRESAHA.122.321690

**Published:** 2023-01-24

**Authors:** Robert Clarke, Adam Von Ende, Lukas E. Schmidt, Xiaoke Yin, Michael Hill, Alun D. Hughes, Raimund Pechlaner, Johann Willeit, Stefan Kiechl, Hugh Watkins, Konstantinos Theofilatos, Jemma C. Hopewell, Manuel Mayr

**Affiliations:** 1 Clinical Trial Service Unit and Epidemiological Studies Unit, Nuffield Department of Population Health, University of Oxford, United Kingdom (R.C., A.V.E., M.H., J.C.H.).; 2 King’s British Heart Foundation Centre, School of Cardiovascular and Metabolic Medicine and Sciences, King’s College London, United Kingdom (L.E.S., X.Y., K.T., M.M.).; 3 MRC Unit for Lifelong Health and Ageing at UCL, Department of Population Science and Experimental Medicine, Institute of Cardiovascular Science, University College London, United Kingdom (A.D.H.).; 4 Department of Neurology, Medical University of Innsbruck, Austria (R.P., J.W., S.K.).; 5 Research Centre on Vascular Ageing and Stroke, Innsbruck, Austria (S.K.).; 6 Division of Cardiovascular Medicine, Radcliffe Department of Medicine, University of Oxford, United Kingdom (H.W.).

**Keywords:** apolipoproteins, cardiovascular diseases, coronary disease, lipids, peptides

## Abstract

Recognition of the importance of conventional lipid measures and the advent of novel lipid-lowering medications have prompted the need for more comprehensive lipid panels to guide use of emerging treatments for the prevention of coronary heart disease (CHD). This report assessed the relevance of 13 apolipoproteins measured using a single mass-spectrometry assay for risk of CHD in the PROCARDIS case-control study of CHD (941 cases/975 controls).

**Methods:**

The associations of apolipoproteins with CHD were assessed after adjustment for established risk factors and correction for statin use. Apolipoproteins were grouped into 4 lipid-related classes [lipoprotein(a), low-density lipoprotein cholesterol, high-density lipoprotein cholesterol, and triglycerides] and their associations with CHD were adjusted for established CHD risk factors and conventional lipids. Analyses of these apolipoproteins in a subset of the ASCOT trial (Anglo-Scandinavian Cardiac Outcomes Trial) were used to assess their within-person variability and to estimate a correction for statin use. The findings in the PROCARDIS study were compared with those for incident cardiovascular disease in the Bruneck prospective study (n=688), including new measurements of Apo(a).

**Results:**

Triglyceride-carrying apolipoproteins (ApoC1, ApoC3, and ApoE) were most strongly associated with the risk of CHD (2- to 3-fold higher odds ratios for top versus bottom quintile) independent of conventional lipid measures. Likewise, ApoB was independently associated with a 2-fold higher odds ratios of CHD. Lipoprotein(a) was measured using peptides from the Apo(a)-kringle repeat and Apo(a)-constant regions, but neither of these associations differed from the association with conventionally measured lipoprotein(a). Among HDL-related apolipoproteins, ApoA4 and ApoM were inversely related to CHD, independent of conventional lipid measures. The disease associations with all apolipoproteins were directionally consistent in the PROCARDIS and Bruneck studies, with the exception of ApoM.

**Conclusions:**

Apolipoproteins were associated with CHD independent of conventional risk factors and lipids, suggesting apolipoproteins could help to identify patients with residual lipid-related risk and guide personalized approaches to CHD risk reduction.

**Novelty and Significance**

**What Is Known?**

- Plasma levels of total cholesterol, low-density lipoprotein cholesterol (LDL-C), high-density lipoprotein cholesterol (HDL-C), and triglycerides are routinely measured to predict the risk of cardiovascular disease (CVD), including coronary heart disease.
- Individuals with high levels of residual risk have a high risk of recurrent CVD events despite maximum LDL-C lowering with standard medical treatments.

**What New Information Does This Article Contribute?**

- Apolipoproteins show independent associations with cardiovascular disease after adjustment for conventional lipids.
- The associations of apolipoproteins with CVD have high reproducibility in both retrospective and prospective studies in independent European populations.
- A single, standardized mass spectrometry assay targeting a comprehensive panel of apolipoproteins affords an alternative to measuring individual apolipoproteins by different immunoassays.

Apolipoproteins constitute the main protein components of lipoproteins. This study examined the associations of conventional lipid measures and 13 related apolipoproteins with the risk of coronary heart disease and wider CVD, before and after adjustment for established risk factors. The findings illustrate the clinical relevance of apolipoprotein profiling in developing increasingly personalized approaches to risk management.

**Meet the First Author, see p 399**

Plasma levels of total cholesterol, low-density lipoprotein cholesterol (LDL-C), high-density lipoprotein cholesterol (HDL-C), and triglycerides are routinely measured to predict the risk of cardiovascular disease (CVD), including coronary heart disease (CHD).^1,2^ However, measurements of apolipoproteins may be related to CHD, and wider CVD, independent of conventional lipids and may be particularly relevant to quantifying residual risk (ie, substantial excess risk of CHD despite intensive medical management with standard treatments) beyond LDL-C.^1–3^ Individuals with high levels of residual risk have a high risk of recurrent atherosclerotic events despite maximum LDL-C lowering, which prompted additional studies investigating associations of lipoprotein(a) [Lp(a)],^4,5^ apolipoprotein B,^6–8^ and triglyceride-rich lipoproteins with CHD after adjustment for conventional lipids and other established risk factors (eg, smoking, hypertension, and diabetes).^9–11^ Direct measurements of apolipoproteins reflect lipoprotein particle concentrations and composition and provide additional information beyond conventional lipids that may help to guide personalized approaches to treatment.^6–9^ Mass spectrometry (MS) assays offer the opportunity to measure large numbers of such apolipoproteins; however, their relevance for risk of CHD has not yet been fully determined.

Higher plasma levels of Lp(a) are associated with higher risk of CHD.^12,13^ Lp(a) particles are a complex of an LDL particle with an additional Apo(a) [apolipoprotein(a)] linked to ApoB-100. Apo(a) contains a variable number of kringle-IV-type 2 repeats, which have prompted concerns about conventional Lp(a) assays.^14^ Novel MS assays that quantify peptides from the Apo(a)-constant region [Apo(a)-CR] at the Apo(a) C-terminus and the Apo(a)-kringle-IV-type 2 repeat region [Apo(a)-KR] and are independent of Apo(a) molecular mass have recently been developed, but their relationships with CHD risk are uncertain. Previous observational studies have underestimated the independent relevance of triglycerides for CHD,^15,16^ but little is known about the independent relationship with CHD of triglyceride-related apolipoproteins (ApoC1, ApoC2, ApoC3, and ApoE). Likewise, the relevance to CHD risk of HDL-related apolipoproteins beyond ApoA1 (ApoA2, ApoA4, ApoD, ApoH, ApoL1, and ApoM) is not well understood. This study examined the associations of conventional lipid measures and related apolipoproteins with the risk of CHD and wider CVD, before and after adjustment for established risk factors.

To examine how apolipoprotein profiling could be used to quantify the residual risk of CHD, using stable isotope-labeled standards, we expanded a targeted mass spectrometry (MS) assay^17^ to include 13 apolipoproteins for the 4 main lipoproteins: (1) Lp(a)-related [Apo(a)-KR, Apo(a)-CR]; (2) LDL-related (ApoB); (3) triglyceride-related (ApoC1, ApoC2, ApoC3, and ApoE); and (4) HDL-related (ApoA1, ApoA2, ApoA4, ApoD, ApoH, ApoL1, and ApoM). The novel assay panel included 2 peptides for Lp(a) to control for kringle-IV-type 2 polymorphisms and one for each of the other apolipoproteins studied.

The aims of the present study were to compare the shape and strength of the associations of plasma levels of 13 apolipoproteins and conventional lipid measures with CHD in the PROCARDIS (Precocious Coronary Artery Disease) case-control study of CHD, involving 941 cases with early-onset CHD and 975 controls.^4^ In addition, we assessed the within-person variability (n=20) and estimated correction factors for statin treatment (n=20 randomly allocated to statins and n=20 to placebo) for all apolipoproteins in the ASCOT trial (Anglo-Scandinavian Cardiac Outcomes Trial),^18,19^ which were subsequently applied in analyses of the PROCARDIS data. In addition to the previously reported associations from the Bruneck prospective study (90 CVD events),^17^ we conducted de novo assays of Apo(a)-CR and Apo(a)-KR and compared the associations of all 13 apolipoproteins [including 2 measures of Apo(a)] with CHD in PROCARDIS with those with incident CVD in the Bruneck study. Thus, this report compared the analyses of apolipoproteins with risk of CHD in a retrospective case-control study (PROCARDIS) and with CVD in a prospective study (Bruneck), in which blood samples for apolipoprotein determinations were collected after and before the onset of disease, respectively.

## Methods

### Data Availability

Summary data and additional analyses that support the findings of this study are available from the corresponding authors upon reasonable request.

An expanded Materials and Methods section is provided in the Supplemental Material.

#### PROCARDIS Case-Control Study of CHD

Participants were recruited from 4 European countries (UK, Italy, Sweden, and Germany) between 2004 and 2008 for a retrospective case-control study of CHD.^4^ Ascertainment criteria for CHD cases included a confirmed diagnosis of the following: myocardial infarction (based on standard ECG and enzyme criteria), acute coronary syndrome, or stable angina with a coronary revascularization procedure (coronary artery bypass graft or coronary angioplasty). In addition, all cases had a sibling with a diagnosis of myocardial infarction or acute coronary syndrome before the age of 66 years. Cases with CHD were identified from secondary care records, with CHD events often occurring several years prior to blood collection (between July 29, 1999 and December 31, 2005). Controls were recruited from the same population as the cases (chiefly from spouses or siblings of spouses of CHD cases) and had no personal or sibling history of CHD. Participants providing blood samples were not asked to fast before doing so, but the time interval since their last meal was recorded. The study procedures were approved by the ethics committee of each participating country, and all participants provided written informed consent.

#### Anglo-Scandinavian Cardiac Outcomes Trial

The ASCOT trial was a double-blind randomized 2×2 factorial trial of blood pressure-lowering and lipid-lowering treatment.^18,19^ A total of 14 412 participants (aged 40–79 years) were randomly allocated to atorvastatin (10 mg/day) or placebo between 1998 and 2000. For the present report, a random sample of 40 individuals (20 allocated to atorvastatin and 20 allocated to placebo) was selected from an age and sex-matched subset in the ASCOT trial with available plasma samples. The within-person variability in apolipoprotein levels was estimated in the placebo-allocated group (n=20) based on the repeat measurements made at baseline and at 1 year after baseline, and the percentage change between the statin group and the placebo group was estimated.

#### Bruneck Prospective Study

The Bruneck study is a community-based prospective study of 1000 men and women from Italy that were enrolled in 1990 and resurveyed every 5 years. The present analysis related to 688 men and women surveyed in 2000 and followed up for incident CVD outcomes (n=90 events) until 2010. De novo laboratory analyses were performed on Apo(a)-CR and Apo(a)-KR in Bruneck and these were added to the apolipoprotein data previously reported by Pechlaner et al.^17^

## Laboratory Methods

The apolipoprotein measurements for PROCARDIS, Bruneck, and ASCOT studies were conducted at King’s College London. MS measurements in the PROCARDIS (in 2018) and Bruneck (in 2014) studies were performed by multiple reaction monitoring, and MS measurements in ASCOT (in 2020) by parallel reaction monitoring. Plasma proteins were denatured, reduced, and alkylated. Alkylated proteins were spiked with AQUA Ultimate stable isotope-labeled standard peptides (Table S1, Thermo Scientific), and an in-solution trypsin digestion was performed overnight. Peptide C18 clean-up was achieved using a Bravo AssayMAP Liquid Handling Platform (Agilent). Cleaned peptide solutions were separated on an AdvanceBio Peptide Mapping Reversed-Phase column (Agilent) and analyzed using dynamic multiple reaction monitoring on a 6495 triple quadrupole mass spectrometer (Agilent), or using parallel reaction monitoring on a Q Exactive HF mass spectrometer (Thermo Scientific). MS raw data were analyzed using SpectroDive (Biognosys) or Skyline.^20^ Apolipoprotein assays were conducted using similar methods for PROCARDIS, Bruneck, and ASCOT studies. A more detailed description of the MS methods is available in the Supplemental Material.

Conventional Lp(a) levels were measured using a latex-enhanced immunoturbidimetric assay (Randox Laboratories, Crumlin, Co. Antrim, UK) based on a highly specific polyclonal rabbit anti-Apo(a) antibody on an ADVIA 1800 autoanalyser (Siemens, Erlangen, Germany) in the Leibniz-Institute for Atherosclerosis at the University of Münster, Münster, Germany. The Lp(a) assays had been validated against the ELISA reference method and used 5 calibrators derived from World Health Organization reference material SRM2B. Other conventional lipid measurements were performed at the Wolfson laboratory, University of Oxford, Oxford, UK. Laboratory analyses of lipid fractions, including HDL-C, directly measured LDL-C and triglycerides were performed using standard methods on automated analyzers. All assays were performed in EDTA plasma samples that had been stored (for up to 10 years) in liquid nitrogen or in −80 °C freezers prior to analysis.

## Statistical Methods

Apolipoprotein levels were measured in 1941 individuals (958 CHD cases and 983 controls) in the PROCARDIS study. After exclusion of individuals with missing data on 1 or more covariates, complete data were available for 941 cases and 975 controls. Apolipoproteins and other lipids in PROCARDIS were corrected for statin medication if, in the ASCOT data (Table S2), the within-person absolute change was statistically significant (*P*<0.05) in the statin-allocated group, but not in the placebo-allocated group (Figure S1). The mean levels of 13 apolipoproteins and conventional lipids (LDL-C, HDL-C, and triglycerides) were corrected for statin use in PROCARDIS, where it is not possible to undertake a standard covariate adjustment for statin use due to complete confounding of statin status by case-control status. The correction was undertaken by multiplying the apolipoprotein concentrations in statin use by an estimated correction factor (Table S3), which was defined as one minus the percentage change between the off-statin (baseline) and on-statin (1 year) measurements based on 20 statin-allocated participants in ASCOT.^18,19^ Baseline characteristics were presented as mean values (SD) and numbers (percentages) unless otherwise indicated. Spearman coefficients were used to assess correlations of apolipoproteins with each other and with conventional lipids. Logistic regression models were used to assess the associations of CHD risk across quintiles (based on controls) or per 1 SD higher unit (calculated in controls) of each apolipoprotein and lipid variable, and were adjusted for established risk factors (age, sex, country, smoking, hypertension, and diabetes) and hours since last meal. Sensitivity analyses examined the associations with CHD after adjustment for body mass index and also examined the associations of apolipoproteins or lipids with CHD risk in participants with no prior history of diabetes. Empirical estimates of variance derived from generalized estimating equations were used to account for familial clustering. Group-specific variances were estimated to enable comparisons between different groups without restriction to a single arbitrary reference group in Figure 2. A Bonferroni correction method was applied to account for multiple testing of the 14 MS measurements, resulting in a conservative *P*-value threshold of <0.05/14=0.0036 used to infer statistical significance. All analyses were performed using SAS v9.3 (Cary, NC, USA) and R v4.1.

**Figure 1.**
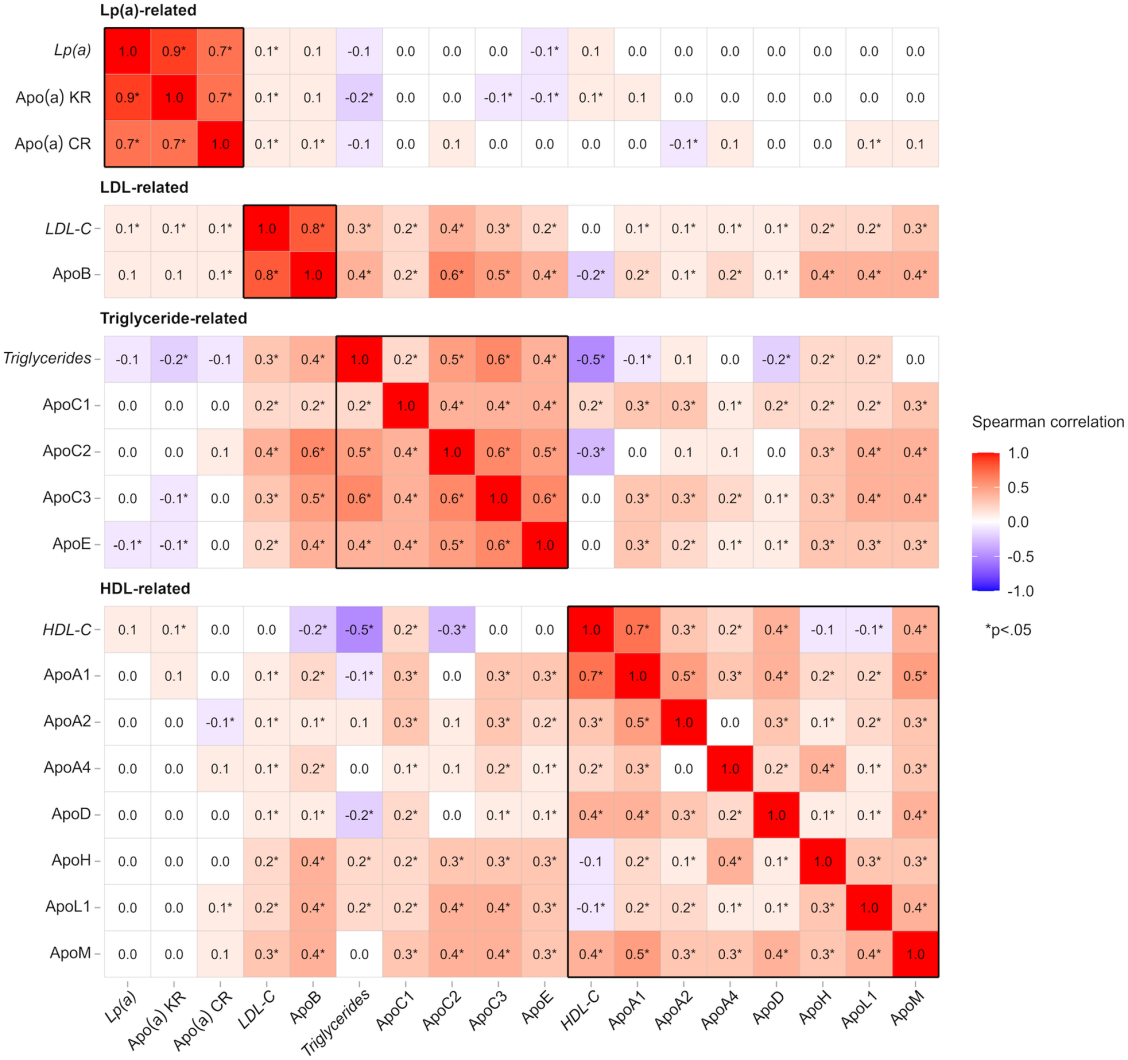
**Spearman correlations between apolipoproteins and conventional lipids in 975 controls in PROCARDIS.** Complete data (n=975) were available for all measurements except Apo(a)-KR (n=972) and Apo(a)-CR (n=122). Lipid values in italics were measured at the Wolfson Laboratory, University of Oxford. The numbers shown are pairwise Spearman correlations. The magnitude of the correlations are illustrated by the color with white being effectively zero or no correlation. Apo(a)-CR indicates Apo(a) constant-region peptide (LFLEPTQADIALLK); Apo(a)-KR, Apo(a) kringle-IV-type 2 repeat peptide (GTYSTTVTGR); HDL-C, high-density lipoprotein cholesterol; LDL-C, low-density lipoprotein cholesterol.

## Results

### Baseline Characteristics of Participants in the PROCARDIS Study

Table 1 shows the baseline characteristics of CHD cases and controls in the PROCARDIS study. Cases and controls were well matched for age (63.0 [6.9] versus 60.9 [10.0] years) and sex (32.3% versus 30.7% females). As expected, CHD cases had a higher proportion of cigarette smokers (10 years prior to recruitment; 41.8% versus 19.8%), diabetes (16.3% versus 1.5%), and hypertension (51.9% versus 23.2%) than controls. Likewise, CHD cases had higher mean (SD) levels of body mass index (28.5 [4.7] versus 26.7 [4.0] kg/m^2^), and a higher proportion of statin users than controls (66.4% versus 0%), respectively. Mean levels of LDL-C (uncorrected for statin use) were lower in CHD cases than in controls (2.8 [0.8] versus 3.3 [0.8] mmol/L), but mean levels of triglycerides were higher in cases than controls (2.2 [1.6] versus 1.7 [1.1] mmol/L), and mean levels of HDL-C were lower in cases than controls (1.1 [0.3] versus 1.4 [0.4] mmol/L).

**Table 1.**
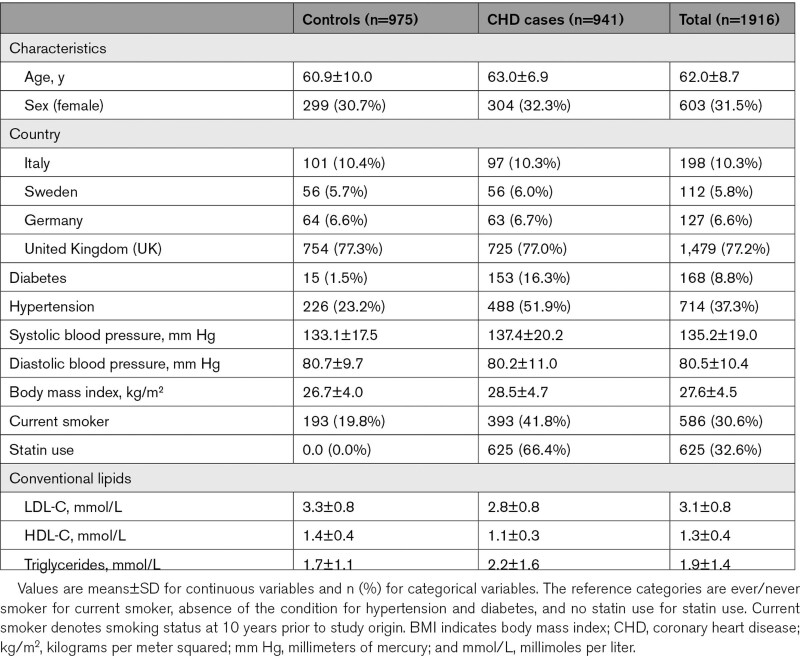
Baseline Characteristics of Controls and CHD Cases in PROCARDIS

### Correlation of Apolipoproteins With Conventional Lipids in PROCARDIS

Figure 1 shows the correlation coefficients for the apolipoproteins with each other and with lipid measurements [Lp(a), LDL-C, triglycerides, and HDL-C] in controls in the PROCARDIS study. Plasma levels of Lp(a) assessed using conventional assays were highly correlated with Apo(a)-KR peptide (r=0.9) and to a lesser degree with the Apo(a)-CR peptide (r=0.7). Likewise, the Apo(a)-CR and Apo(a)-KR peptides were also correlated with each other (r=0.7), but not with any of the other apolipoproteins studied. ApoB was highly correlated with LDL-C (r=0.8) assessed using a conventional direct assay and modestly correlated with ApoC2 (r=0.6) and ApoC3 (r=0.5), but was only weakly correlated with the other apolipoproteins studied. Triglycerides were positively correlated with ApoC2 (r=0.5) and ApoC3 (r=0.6), and were inversely correlated with HDL-C (r=-0.5). ApoC2 levels were modestly correlated with ApoC3 (r=0.6) and ApoE (r=0.5). ApoA1 was correlated with HDL-C (r=0.7), and modestly correlated with ApoA2 (r=0.5). Neither ApoD, ApoH, ApoL1 nor ApoM were correlated with each other or with any of the other apolipoproteins studied.

### Within-Person Variability of Apolipoproteins and Effect of Statin Therapy in ASCOT

The baseline characteristics of the ASCOT trial participants are shown in Table S2. Table 2 shows median plasma levels, within-person SD, between-person SD, and self-correlation coefficients for the apolipoproteins among 20 placebo-allocated individuals in the ASCOT trial. The within-person SDs (intra-assay) were about half those of the between-person SDs (interassay) for most apolipoproteins. The self-correlations were high (r≥0.80) for Apo(a)-KR, Apo(a)-CR, ApoA4, ApoE, ApoH, and ApoL1, moderate (≥0.60) for ApoA1, ApoA2, ApoB, ApoC2, ApoC3, ApoD, and ApoM, and low (<0.50) for ApoC1.

**Table 2.**
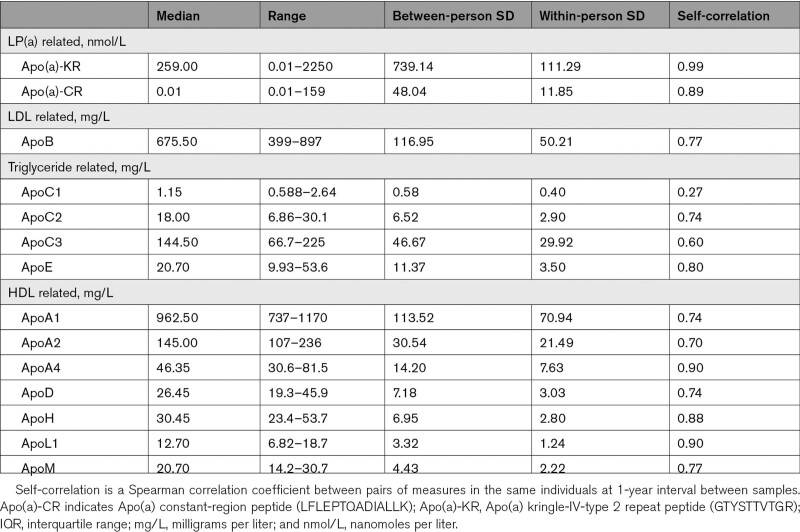
Distribution and Between- and Within-Person Variability of 13 Apolipoproteins in 20 Placebo Participants in the ASCOT Trial

Complete data on Apo(a)-KR were available on 1913 participants in PROCARDIS (941 cases/972 controls), but complete data on Apo(a)-CR were only available on 346 of these (224 cases/122 controls) as the remaining Apo(a)-CR values (80%) were below the limit of detection by targeted MS.

Similarly, <80% of Apo(a)-CR values in the analyzed ASCOT participants (20 on statins/20 on placebo) were below the limit of detection.

**Figure 2.**
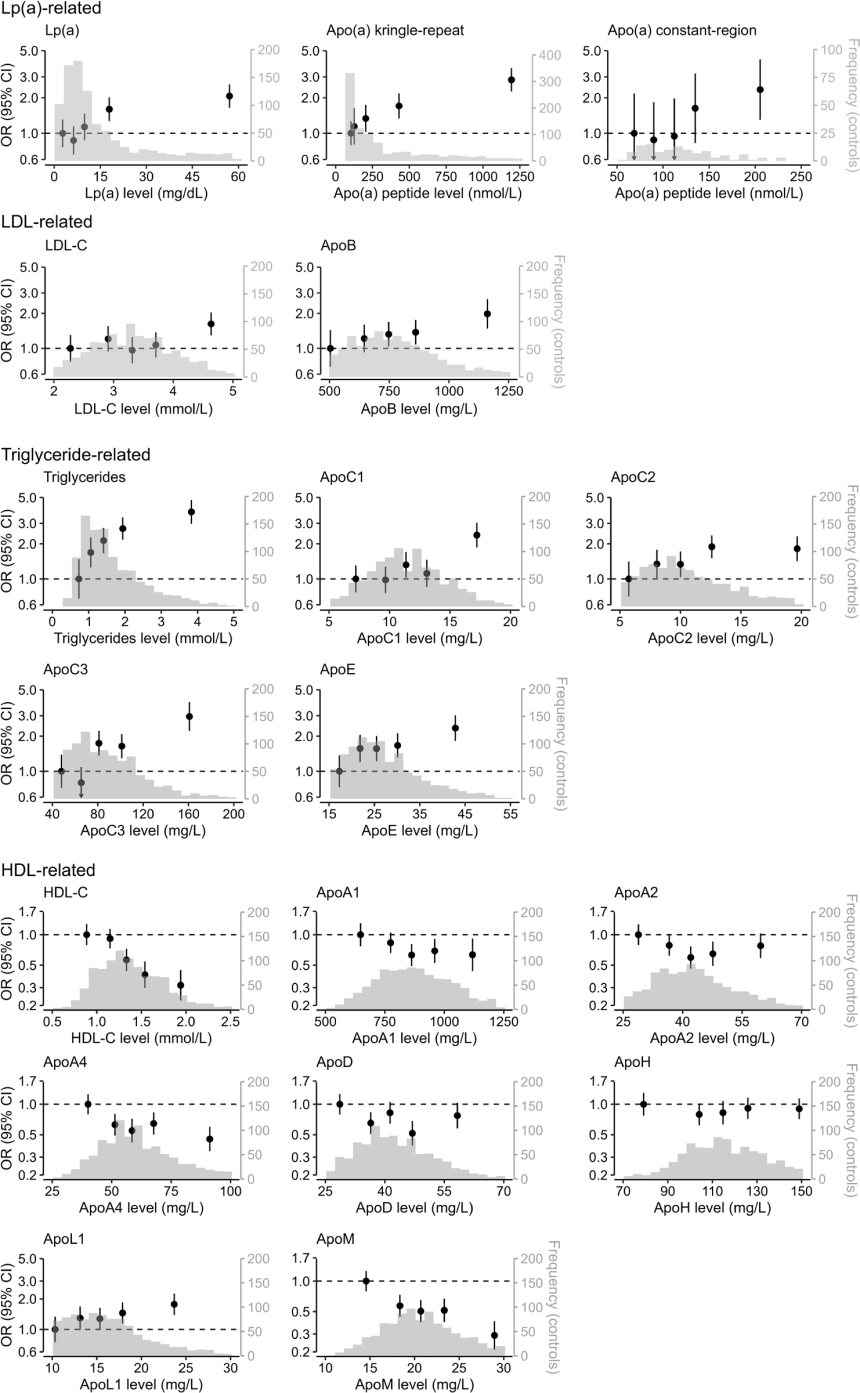
**Distribution of apolipoproteins and conventional lipids in PROCARDIS controls, and risk of CHD by quintiles among 941 CHD cases and 975 controls.** Complete data were available for each biomarker on 941 cases and 975 controls, with the exception of Apo(a) kringle-repeat (941 cases/972 controls) and Apo(a) constant-region (224 cases/122 controls). The histograms were generated in controls. All odds ratios were adjusted for age, sex, country, smoking, diabetes, hypertension, and hours since last meal. The 95% CIs were estimated using floating variances. Apo indicates apolipoprotein; CHD, coronary heart disease; HDL-C, high-density lipoprotein cholesterol; LDL-C, low-density lipoprotein cholesterol; Lp(a), lipoprotein(a); mg/dL, milligrams per deciliter; mg/L, milligrams per liter; mmol/L, millimoles per liter; nmol/L, nanomoles per liter; and OR, odds ratio.

Figure 2 shows the distributions of each of the apolipoproteins in controls, and the associations across quintiles (after correction for statin use where appropriate) with risk of CHD after adjustment for established CHD risk factors (age, sex, country, smoking, hypertension, and diabetes) and hours since last meal. Table S4 shows the distribution of cases and controls, cutoff points, and median values by quintiles for each of the lipids and apolipoproteins studied. While the distributions of Apo(a)-CR and Apo(a)-KR peptides were highly skewed, the other apolipoproteins were approximately normally distributed.

### Associations of Apolipoproteins With Risk of CHD in PROCARDIS

**Figure 3.**
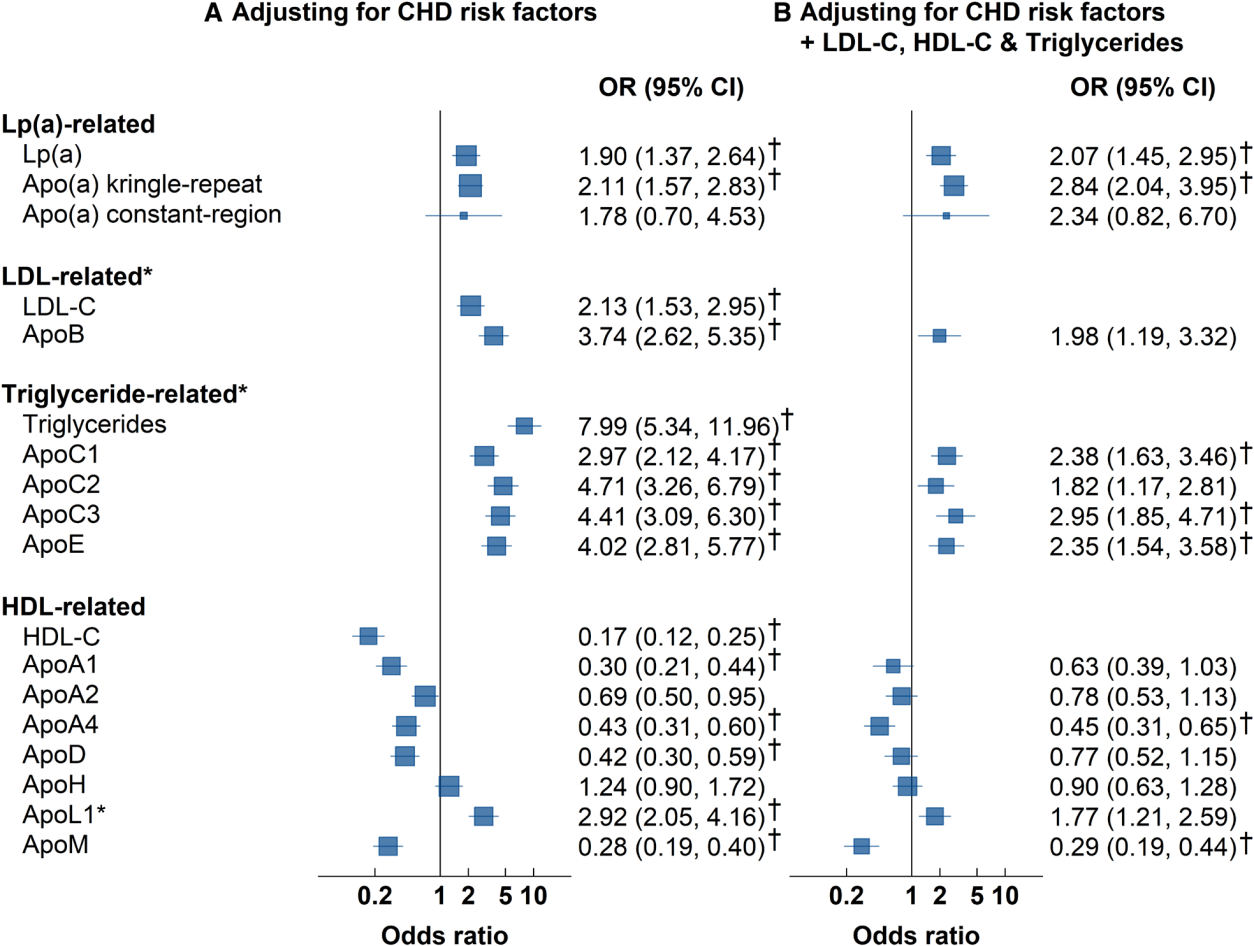
**Risk of CHD in top versus bottom quintile of apolipoproteins before and after adjustment for conventional lipids, among 941 CHD cases and 975 controls in PROCARDIS. A**, This shows the ORs of CHD after adjustment for age, sex, country, smoking, diabetes, hypertension, and hours since last meal. **B**, This shows the ORs of CHD after additional adjustment for conventional lipids. Complete data were available for all measurements in 941 cases and 975 controls, with the exception of Apo(a)-KR (941 cases/972 controls) and Apo(a)-CR (224 cases/122 controls). *P*-values derived from likelihood ratio tests comparing models with and without the 5-category apolipoprotein variable. *Corrected for statin use; †Survived multiple testing (Bonferroni-adjusted alpha=0.0036 [0.05/14]). Apo indicates apolipoprotein; CHD, coronary heart disease; HDL-C, high-density lipoprotein cholesterol; LDL-C, low-density lipoprotein cholesterol; Lp(a), lipoprotein(a); and OR, odds ratio.

The shapes of the associations with risk of CHD for most of the apolipoproteins were approximately log-linear throughout the ranges studied. Figure 3 provides a comparison of the odds ratio (OR) of CHD for top versus bottom quintile of plasma levels for each apolipoprotein after adjustment for established CHD risk factors and conventional lipids. Higher levels of conventional measures of Lp(a), and both MS measures of Apo(a)-KR and Apo(a)-CR peptides, were equally strongly and positively associated with CHD.

The results demonstrated slightly stronger associations with CHD for top versus bottom quintile of ApoB compared with conventional LDL-C measures (OR [95% CI], 3.74 [2.62–5.35] versus 2.13 [1.53–2.95]; Figure 3). After adjusting for conventional lipids including LDL-C, the OR (95% CI) of CHD for ApoB was 1.98 (1.19–3.32). Likewise, higher levels of apolipoproteins related to triglyceride-rich lipoproteins, including ApoC1, ApoC3, and ApoE were associated with CHD after adjustment for other conventional lipids and correction for multiple testing. However, the association of ApoC2 was no longer statistically significant after correction for multiple testing. Among the HDL-related lipids, the associations of higher levels of ApoA4 and ApoM were both inversely associated with CHD after adjusting for HDL-C and other conventional lipid measurements. In contrast, higher levels of ApoL1 were positively associated with CHD, although this was not significant after correction for multiple testing. Figure S2 shows the associations of extreme quintiles of apolipoproteins with CHD with and without correction for statin treatment in PROCARDIS. Table S5 shows that the associations of apolipoproteins with CHD were unaltered by additional adjustment for body mass index.

### Stratification by Presence of Diabetes

Table S6 compares the OR (95% CI) of CHD in PROCARDIS for apolipoproteins and conventional lipids in all participants (n=1916) and a subset excluding individuals with type 2 diabetes (n=1748). The results demonstrated no material differences in the strength of associations of apolipoproteins or conventional lipids with CHD in all individuals and a subset without diabetes.

### Comparisons With the Bruneck Prospective Study

**Figure 4.**
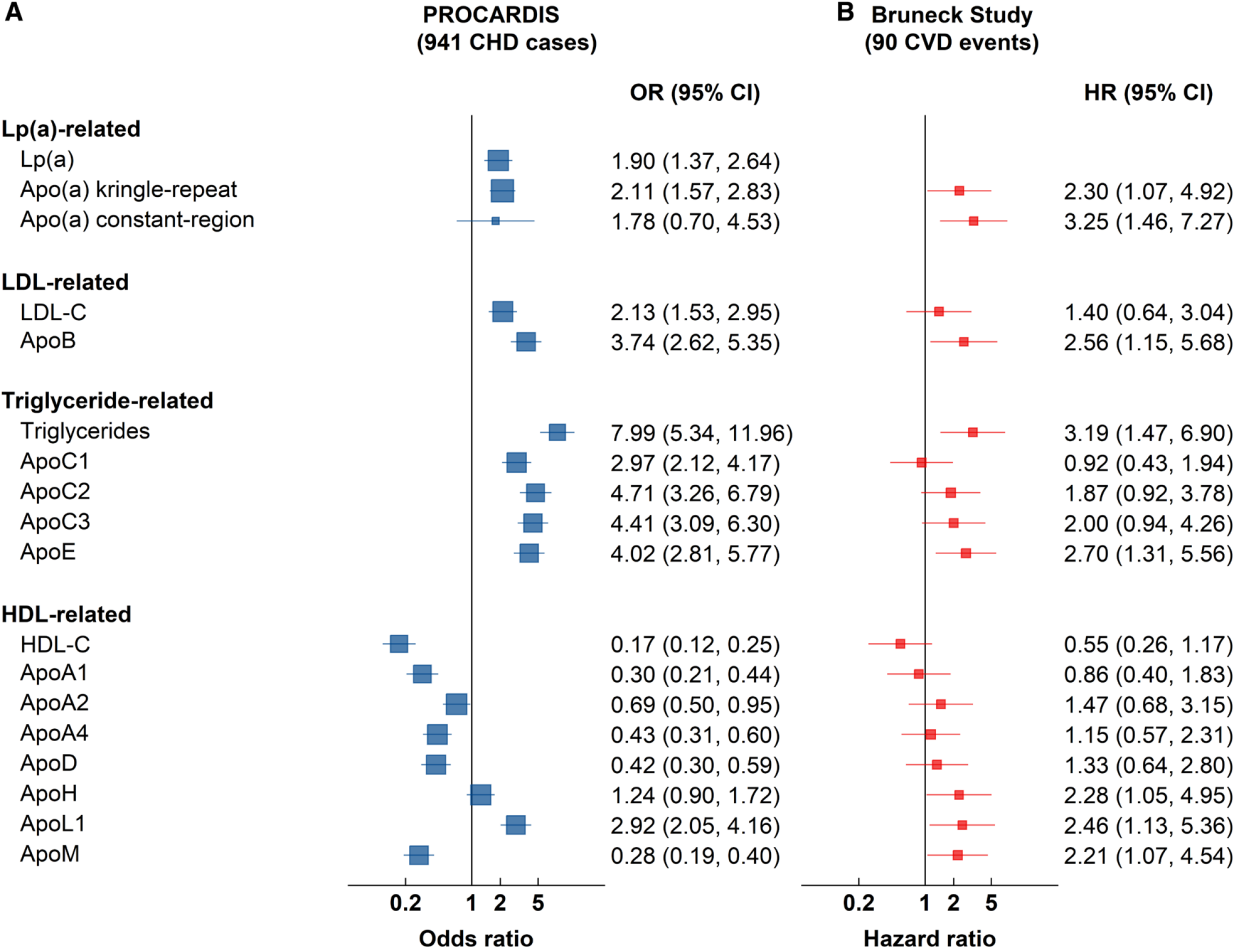
**Risk of CHD in PROCARDIS and of CVD in Bruneck, in top versus bottom quintile of apolipoproteins and conventional lipids.** Complete data were available for all measurements in 941 cases and 975 controls in PROCARDIS, with the exception of Apo(a)-KR (941 cases/972 controls) and Apo(a)-CR (224 cases/122 controls). PROCARDIS estimates are adjusted for age, sex, country, smoking, diabetes, hypertension, and hours since last meal. Participant level values for LDL-C, ApoB, triglycerides, ApoC1, ApoC2, ApoC3, ApoE, and ApoL1 are corrected for statin use. Complete data were available in the Bruneck study, with the exception of Apo(a)-CR: 19/90 (21.1%) participants with CVD events and 89/598 (14.9%) participants without CVD events had Apo(a)-CR above limit of detection. Bruneck estimates are adjusted for age, sex, smoking, diabetes, hypertension, and statin use. Boxes represent effect sizes with their size inversely proportional to variance. Apo indicates apolipoprotein; CHD, coronary heart disease; CVD, cardiovascular disease; HDL-C, high-density lipoprotein cholesterol; HR, hazard ratio; LDL-C, low-density lipoprotein cholesterol; Lp(a), lipoprotein(a); and OR, odds ratio.

The associations of all the apolipoproteins with CHD studied in the PROCARDIS case-control study, after adjustment for established CHD risk factors, were compared with associations with incident CVD events (n=90) in a 10-year follow-up of a subset of 688 individuals in the Bruneck study. The apolipoproteins in Bruneck were measured on stored plasma samples collected in the year 2000 evaluation.^17^ For this comparison, we conducted de novo assays of Apo(a)-CR and Apo(a)-KR in the Bruneck study. About 85% of individuals without subsequent CVD and 79% of CVD cases had Apo(a)-CR below the lower limit of detection in Bruneck. Baseline characteristics of participants in the Bruneck study are shown in Table S7. Figure 4 compares the ORs (95% CI) of CHD for top versus bottom quintile of apolipoproteins and conventional lipids after adjustment for established risk factors in the PROCARDIS and Bruneck studies. Additional analyses of CHD associations for a 1 SD difference in measures are also provided to compare with previous results (Figure S3). Moreover, the results demonstrated that 9 of the 14 MS measurements were directionally consistent and showed no heterogeneity in effect size (*P*_het_<0.05) between the PROCARDIS and Bruneck studies. Among the 7 MS measurements whose associations were robust to multiple testing in PROCARDIS, 4 [Apo(a)-KR, Apo(a)-CR, ApoC3, and ApoE] showed no evidence of heterogeneity when compared to the estimates in the Bruneck study (*P*_het_>0.05). Furthermore, apolipoproteins (ApoC2 and ApoL1) with suggestive associations (*P*<0.05 before multiple testing corrections) were directionally consistent with Bruneck. Overall, apart from ApoM, which was inversely associated with CHD in PROCARDIS but positively associated with CVD in Bruneck, the associations with all other apolipoproteins were directionally consistent in both studies.

## Discussion

There is an unmet clinical need for novel assays that measure key apolipoproteins to aid in quantifying residual lipid-related risk of CHD (after statin therapy). The European Society of Cardiology Guidelines on the Management of Dyslipidaemia currently advocate measurements of Lp(a) and ApoB in addition to LDL-C, HDL-C, and triglycerides and acknowledge that LDL-C, non-HDL-C, and ApoB provide essentially similar information, but plasma levels of LDL-C and ApoB can be discordant in some settings. Importantly, the present report highlights the independent relevance of triglyceride-related apolipoproteins for CHD after adjusting for conventional lipids. This suggests that mechanisms by which plasma triglycerides are related to CHD risk might be mediated by the composition and concentration of lipoprotein particles that transport them.

### Analysis of Apolipoproteins in PROCARDIS

Among the 13 apolipoproteins studied in PROCARDIS, the associations of triglyceride-related apolipoproteins ApoC1, ApoC3, and ApoE were strongly and positively associated with risk of CHD after adjustment for triglycerides and other conventional lipid measures and correction for statin treatment. Among the HDL-related apolipoproteins, ApoA4 and ApoM were strongly and inversely associated with CHD after adjustment for HDL-C and other conventional lipid measures and correction for statin treatment. The associations of both Apo(a)-CR and Apo(a)-KR peptides with CHD were comparable with those based on conventional Lp(a) measures. Thus, this study demonstrated independent associations of apolipoproteins with CHD, and also with CVD, with high reproducibility in both retrospective and prospective studies in independent European populations.

### Independence Relevance Over Conventional Lipid Measures

While the associations of ApoB with CHD were stronger than those for LDL-C in PROCARDIS, the associations of ApoB with CHD were also independent of LDL-C. The strength of the associations of ApoB with CHD in PROCARDIS were comparable with those with CVD in the Bruneck study and with previous studies.^6–8,17^ The associations of the triglyceride-related apolipoproteins with CHD in PROCARDIS were also consistent with the Bruneck study, but the 10-fold larger number of cases in PROCARDIS confirmed the previously reported findings and demonstrated their independence from conventional lipids. The PROCARDIS study also revealed strong positive associations of ApoC1, ApoC3, and ApoE with CHD independent of triglycerides. In contrast to ApoC3, ApoC2 is an activator of lipoprotein lipase and the association of ApoC2 with CHD in PROCARDIS was not statistically significant after correction for multiple testing.

### CHD and HDL-Related Apolipoproteins

Among HDL-related proteins, the strength of the associations of HDL-C with CHD was more extreme in PROCARDIS than in the Bruneck study.^17^ Most HDL-related proteins, except for ApoL1 and ApoH, were inversely associated with CHD in PROCARDIS, but only ApoA4 and ApoM were associated with CHD after adjusting for HDL and other conventional lipid measures. Previous observational studies had reported inverse associations of ApoA4 with CVD,^21,22^ but the available evidence on the role of ApoA4 for risk of CHD is limited. The results highlight the independent relevance of HDL-associated apolipoproteins after adjustment for HDL-C. In contrast with other HDL-related apolipoproteins, which are inversely associated with CHD, higher levels of ApoL1 were positively associated with CHD in PROCARDIS, consistent with the role of ApoL1 in inflammation.^23–25^ ApoM was inversely associated with CHD in PROCARDIS, but not in the Bruneck study.^17^ ApoM is bound primarily to HDL, where it facilitates the formation of preβ-HDL, and plays a role in glucose homeostasis and dyslipidaemia of diabetes.^26^ Neither ApoD nor ApoH were associated with CHD in PROCARDIS after adjustment for established CHD risk factors, HDL-C, and other conventional lipid measures.

### MS for Precision Medicine

Recent technological advances in high-throughput MS allow for simultaneous quantification of a comprehensive panel of apolipoproteins in large-scale studies. Previous studies evaluating MS assays of apolipoproteins with risk of CVD involved only a few apolipoproteins or included only a modest number of CVD cases and controls.^26–31^ Two studies evaluated 6 apolipoproteins (ApoA1, ApoB, ApoC1, ApoC2, ApoC3, and ApoE) with venous thromboembolism (n=426)^24^ or acute myocardial infarction (n=519).^30^ Other studies evaluated several apolipoproteins with coronary, peripheral artery disease, or cerebral vascular disease (n=911).^21^ The largest study to date (n=1468) evaluated 4 apolipoproteins [Apo(a), ApoA1, ApoA2, and ApoB] with CVD in patients with diabetes.^32^ The present study involved a larger sample size (n=1916) and measured 13 apolipoproteins in nonfasting plasma samples and highlighted the independent relevance of triglyceride-related apolipoproteins for CHD after adjustment for conventional lipids. The findings provide support for therapeutic targets for remnant cholesterol that have been linked to CVD beyond LDL-C and ApoB.^33^ The International Federation of Clinical Chemistry Working Group for Standardisation of Apolipoproteins by MS conceptualized a reference measurement system for 7 apolipoproteins [Apo(a), ApoA1, ApoB, ApoC1, ApoC2, ApoC3, and ApoE] to facilitate standardization of these assays in reference laboratories.^34^ The relevance of additional apolipoproteins (ApoA2, ApoA4, ApoD, ApoH, ApoL1, or ApoM) is not yet fully understood, but some show associations with CHD in the present study. Using a single, standardized MS assay, we measured a panel of 13 apolipoproteins as an alternative approach to measuring individual apolipoproteins by different immunoassays.^34^ A key differentiator is that apolipoproteins were measured directly by MS without the need for binders. Peptide measurements are expected to be independent of the lipid content or size of lipoproteins and by using stable isotope-labeled standards, levels of different apolipoproteins can be compared directly. Moreover, intersample variation by MS is low particularly for standard flow applications for higher throughput.^35^

### Apolipoproteins as Novel Therapeutic Targets

The advent of novel lipid-modifying agents beyond LDL-C-lowering for prevention of CHD has prompted interest in Lp(a) and other apolipoprotein assays to facilitate targeted treatment for residual lipid-related risk in patients at high risk of CHD.^17,36,37^ Pelacarsen (Ionis Pharmaceuticals) is an antisense oligonucleotide designed to reduce Lp(a). Volanesorsen (Ionis Pharmaceuticals) is an antisense oligonucleotide that inhibits hepatic synthesis of ApoC3 and used for the treatment of familial chylomicronemia syndrome, familial partial lipodystrophy, and hypertriglyceridemia. Several large trials are currently assessing the efficacy of these antisense oligonucleotide agents for the prevention of CHD.^36,37^ Volanesorsen lowers ApoC3 (mean decreases >75%), but also reduces ApoC2 and ApoE (mean decreases ~50%) as demonstrated by our MS assay.^16^ Thus, the availability of new therapeutics for lipid-lowering has also reinforced the need to provide more comprehensive apolipoprotein assays to inform treatment decisions.

### Limitations

The PROCARDIS study had several limitations, including a retrospective study design, use of nonfasting blood samples, and high degree of statin use among CHD cases. Nevertheless, the findings observed in PROCARDIS were directionally consistent with those in the Bruneck prospective study, which used fasting blood samples for analyses and controlled for statin use. Despite differences in the disease outcomes (CHD in PROCARDIS and a composite CVD endpoint [myocardial infarction, stroke, and sudden cardiac death] in Bruneck) between studies in the present report, the independent relevance of these apolipoproteins for occlusive vascular diseases in both studies was remarkably consistent. Also, while the PROCARDIS study analyzed the independent associations of apolipoprotein concentrations with CHD after adjusting for conventional lipids, these associations cannot include or exclude possible interactions of these apolipoproteins with each other,^38^ which should be a direction of future work. Finally, we do not provide additional sensitivity analyses stratified by statin therapy, as such analyses are likely to be highly susceptible to selection bias, whereby patients with greater disease severity or with higher LDL levels were more likely to be prescribed statins at enrollment to this study and any such stratified analyses by treatment may result in spurious differences in subgroup analyses by statin treatment.

## Conclusions

Currently, apolipoproteins (beyond ApoB or ApoA1) are not routinely measured when estimating risk of CHD, or wider CVD, but there is an unmet clinical need for more comprehensive apolipoprotein assays. The findings of this study highlight the utility of a single MS assay measuring 13 apolipoproteins and the potential value of apolipoprotein profiling in developing increasingly personalized approaches to risk management, and guiding the use of lipid-modifying medications beyond statin therapy that may further reduce the risk of CHD in patients with optimized LDL-C levels.

## Article Information

### Sources of Funding

The PROCARDIS study was funded by 6th Framework Programme of the European Union (LSH-2005-2.1.1.1), British Heart Foundation, and Astra Zeneca. R. Clarke, H. Watkins, and J. Hopewell acknowledge support by the British Heart Foundation Centre for Research Excellence, Oxford. J. Hopewell was funded by the British Heart Foundation (FS/14/55/30806) and also acknowledges support from the National Institute for Health Research Oxford Biomedical Research Centre, and the Nuffield Department of Population Health, University of Oxford, UK. M. Mayr is a British Heart Foundation (BHF) Chair Holder (CH/16/3/32406) with BHF programme grant support (RG/F/21/110053). K. Theofilatos and M. Mayr were also supported by a BHF project grant (PG/20/10387). The research was also supported by the National Institute for Health Research (NIHR) Biomedical Research Centre based at Guy’s and St Thomas’ NHS Foundation Trust and King’s College London (the views expressed are those of the author(s) and not necessarily those of the NHS, the NIHR, or the Department of Health). The Bruneck Study is supported by the Pustertaler Verein zur Vorbeugung von Herz- und Hirngefässerkrankungen, the Gesundheitsbezirk Bruneck, and the Sanitätsbetrieb Südtirol, province of Bolzano, Italy, and received support from the excellence initiative (Competence Centers for Excellent Technologies – COMET) of the Austrian Research Promotion Agency FFG (K-Project No. 843536) funded by the BMVIT, BMWFW, Wirtschaftsagentur Wien, Wirtschafts- und Forschungsförderung Salzburg and Standortagentur Tirol. M. Mayr is also supported by the Leducq Foundation (18CVD02) and VASCage-C (Research Centre on Vascular Ageing and Stroke), an R&D K-Centre of the Austrian Research Promotion Agency (COMET program—Competence Centres for Excellent Technologies) funded by the Austrian Ministry for Transport, Innovation, and Technology, the Austrian Ministry for Digital and Economic Affairs and the federal states Tyrol, Salzburg, and Vienna with the grant number FSG 868624. ASCOT was funded by major grants from Pfizer to Imperial College London and to the Gothenberg Trial Centre. Additional support for ASCOT was received from Laboratoire Servier and Solvay Pharmaceuticals. A. Hughes receives support from the British Heart Foundation, the Horizon 2020 Framework Programme of the European Union, the National Institute for Health Research University College London Hospitals Biomedical Research Centre, the UK Medical Research Council, the Wellcome Trust, and works in a unit that receives support from the UK Medical Research Council.

### Disclosures

The Clinical Trial Service Unit and Epidemiological Studies Unit, Nuffield Department of Population Health receives research grants from industry that are governed by University of Oxford contracts that protect its independence, and has a staff policy of not taking personal payments from industry (https://www.ndph.ox.ac.uk/about/independence-of-research). J. Hopewell is a member of various academically-led steering committees for clinical trials of lipid-modifying treatments. M. Mayr is named inventor on patent applications filed by King’s College London. The other authors report no conflicts.

### Supplemental Material

Major Resources Table

Tables S1–S7

Figures S1–S3

## Supplementary Material
