# Supplementary material for: Apolipoprotein Proteomics for Residual Lipid-Related Risk in Coronary Heart Disease
Source: Circ Res. 2023 Jan 24;132(4):452–64. doi: 10.1161/CIRCRESAHA.122.321690 (PMC9930889; doi:10.1161/CIRCRESAHA.122.321690)
Supplement: Supplementary file 1 [file res-132-452-s001.pdf]

SUPPLEMENTAL MATERIAL

Apolipoprotein Proteomics for Residual Lipid-related Risk in Coronary Heart Disease

Members of the PROCARDIS Consortium 2
Expanded Materials and Methods........ 3
Table S1: Stable isotope-labelled standards (SIS) 6
Table S2: Baseline characteristics of the ASCOT trial participants, by allocation to statin treatment ... 7
Table S3: Mean values of apolipoproteins and conventional lipids before and after statin therapy, and percentage change after statin therapy in 20 participants allocated to statin treatment in the ASCOT trial 8
Table S4: Number of cases and controls, cutoff points and median values for quintiles of apolipoproteins and conventional lipids in PROCARDIS 9
Table S5: Comparison of risk of CHD for top versus bottom quintiles of apolipoproteins and conventional lipids adjusting for CHD risk factors and conventional lipids vs adjusting for CHD risk factors and conventional lipids and BMI........ 11
Table S6: Comparison of risk of CHD for top versus bottom quintiles of apolipoproteins and conventional lipids for all participants in PROCARDIS and a subset without diabetes mellitus 12
Table S7: Baseline characteristics of the Bruneck study participants........ 13
Figure S1: Mean plasma concentrations of apolipoproteins and conventional lipids at baseline and one year in ASCOT, by statin allocation 14
Figure S2: Comparison of CHD risk for top versus bottom quintiles of apolipoproteins and conventional lipids, before and after correction for statin use in PROCARDIS........ 15
Figure S3: Comparison of risk of CHD for a one standard deviation difference in apolipoproteins and conventional lipids in PROCARDIS and Bruneck 16

Members of the PROCARDIS Consortium

Department of Cardiovascular Medicine, The Wellcome Trust Centre for Human Genetics, University of Oxford, Oxford, UK. Hugh Watkins (Chair), Martin Farrall, Anuj Goel, Chris Grace, John Peden, Theodosios Kyriakou.

Clinical Trial Service Unit, University of Oxford, Oxford, UK: Robert Clarke, Jemma C Hopewell, Michael Hill, Rory Collins.

Department of Medicine, Atherosclerosis Research Unit, Karolinska Institutet, Karolinska University Hospital, Stockholm, Sweden: Anders Hamsten, John Öhrvik, Per Eriksson, Angela Silveira, Rona Strawbridge, Maria Sabater Lleal

Gesellschaft für Arterioskleroseforschung e.V., Leibniz-Institut für Arterioskleroseforschung an der Universität Münster (LIFA), Münster, Germany: Udo Seedorf, Gerd Assmann.

Department of Cardiovascular Research, Istituto Mario Negri, Milano, Italy: Simona Barlera, Gianni Tognoni, Maria Grazia Franzosi.

Consorzio Mario Negri Sud, Santa Maria Imbaro (Chieti), Italy: Gianni Tognoni.

Biochemical Sciences Division, Faculty of Health and Medical Sciences, University of Surrey, Guilford, UK: Fiona R Green.

Expanded Materials and Methods

In-solution protein digestion and peptide clean-up. 10 µL of plasma were denatured using urea (final concentration 7.2 M) and reduced using dithiothreitol (final concentration 5 mM) for 1 h at 37°C. Reduced proteins were cooled down to room temperature before being alkylated in the dark for 1 h using iodoacetamide (final concentration 25 mM). An aliquot equivalent to about 20 µg of alkylated protein was added to a 0.1 M ammonium bicarbonate solution containing 10 µL of a mix of stable isotope-labeled standard (SIS) peptides (Thermo Scientific, AQUA Ultimate) and digested for 18 h at 37°C using 0.4 µg of Trypsin (Thermo Scientific, 90057) or Trypsin/LysC (Promega, V5072). Digested peptide solutions were acidified using trifluoroacetic acid (TFA, final concentration 1%). Peptide clean-up was done using a Bravo AssayMAP Liquid Handling Platform (Agilent). After conditioning and equilibration of the resin, acidified peptide solutions were loaded onto AssayMAP C18 cartridges (Agilent, 5190-6532), washed using 1% acetonitrile (ACN), 0.1% TFA (aq) and eluted using 70% ACN, 0.1% TFA (aq). Eluted peptides were vacuum centrifuged (Thermo Scientific, Savant SPD131DDA) and resuspended in 40 µL of 2% ACN, 0.05% TFA (aq).

Analysis by multiple reaction monitoring (MRM)–mass spectrometry (MS). Dynamic MRM was used to analyze PROCARDIS samples. 10 µL of peptide solution were assayed using a high-performance liquid chromatography (HPLC)–MS setup consisting of a 1290 Infinity II HPLC system coupled via a Jet Stream Electrospray Ionization source to a 6495 Triple Quadrupole mass spectrometer (all Agilent). At a flow rate of 0.35 mL/min, peptides were separated on an AdvanceBio Peptide Mapping column (Agilent, 651750-902, at 50 °C) using the following gradient: 0–0.1 min, 5–10% B; 0.1–8 min, 10–30% B; 8–9 min, 30–80% B; 9–11 min, 80% B; 11–11.2 min, 80–5% B; 11.2–15 min, 5% B. Mobile phase A was 0.1% formic acid (FA, aq), mobile phase B was 0.1% FA in ACN.

Analysis by parallel reaction monitoring (PRM)–MS. ASCOT samples were analyzed by PRM on an Orbitrap mass analyzer (MS^2 resolution 15,000 at 200 m/z). 10 µL of peptide solution were assayed using a HPLC–MS setup consisting of a Vanquish HPLC system coupled via an Ion Max ion source with a HESI-II probe to a Q Exactive HF mass spectrometer (all Thermo Scientific). At a flow rate of 0.35 mL/min, peptides were separated on an AdvanceBio Peptide Mapping column (same as above, at 50°C) using the following gradient: 0–0.1 min, 5–10% B; 0.1–9 min, 10–32.5% B; 9–10 min, 32.5–80% B; 10–12 min, 80 % B; 12–12.2 min, 80–5% B; 12.2–15 min, 5% B. Mobile phases A and B as above.

MS raw data analysis. MRM data were analyzed in SpectroDive (Biognosys, version 9.10.191220.41419). PRM data were analyzed in Skyline (MacCoss Lab, University of Washington, version 4.2.0.19072). Quantification was based on the peak area sum of all transitions that had been selected for quantification. Using the known amount of SIS, light-to-heavy ratios and absolute molarity concentrations of each peptide were calculated. For all proteins except Apo(a), peptide-based protein mass concentrations in the original plasma samples were calculated from peptide molarities.

Major Resources Table

Antibodies

Target antigen	Vendor or Source	Catalog #	Persistent ID / URL
Lp(a)	Randox Laboratories	Lp(a) was measured in PROCARDIS in 1996	https://randox.com

Chemicals

Name	Vendor	Catalog #	Persistent ID / URL
Acetonitrile with 0.1% Formic Acid (v/v), Optima LC-MS grade	Fisher Chemical	10678935	https://www.fishersci.co.uk/shop/products/acetonitrile-0-1-formic-acid-v-v-optima-lc-ms-grade-thermo-scientific/10678935
Acetonitrile, Optima LC-MS grade	Fisher Chemical	10001334	https://www.fishersci.co.uk/shop/products/acetonitrile-optima-lc-ms-grade-fisher-chemical/10001334
DL-Dithiothreitol	Sigma-Aldrich	D9779	https://www.sigmaaldrich.com/GB/en/product/sigma/d9779
Iodoacetamide	Sigma-Aldrich	I6125	https://www.sigmaaldrich.com/GB/en/product/sigma/i6125
Trifluoroacetic acid, Pierce LC-MS grade	Thermo Scientific	85183	https://www.thermofisher.com/order/catalog/product/85183
Urea	Alfa Aesar	36428	https://www.alfa.com/en/catalog/036428/
Water with 0.1% Formic Acid (v/v), Optima LC-MS grade	Fisher Chemical	10188164	https://www.fishersci.co.uk/shop/products/water-0-1-formic-acid-v-v-optima-lc-ms-grade-thermo-scientific/10188164

Chromatography column

Name	Vendor	Catalog #	Persistent ID / URL
AdvanceBio Peptide Mapping 120Å, 2.1 x 250 mm, 2.7 µm	Agilent	651750-902	https://www.agilent.com/store/ko_KR/Prod-651750-902/651750-902

Consumables

Name	Vendor	Catalog #	Persistent ID / URL
AssayMAP 5 µL C18 cartridges rack of 96	Agilent	5190-6532	https://www.agilent.com/store/en_US/Prod-5190-6532/5190-6532

Enzymes

Name	Vendor	Catalog #	Persistent ID / URL
Trypsin Protease, MS Grade	Thermo Scientific	90057	https://www.thermofisher.com/order/catalog/product/90057
Trypsin/Lys-C Mix, Mass Spec Grade	Promega	V5072	https://www.promega.co.uk/products/mass-spectrometry/trypsin/trypsin-lys-c-mix-mass-spec-grade/?catNum=V5072

Stable-isotope labelled standards

Product	Grade	Vendor	Sequences	Persistent ID / URL
HeavyPeptide AQUA	Ultimate	Thermo Scientific	As described in Table S1	https://www.thermofisher.com/uk/en/home/life-science/protein-biology/peptides-proteins/custom-peptide-synthesis-services/heavypeptide-aqua-custom-synthesis-service.html

Data & Code Availability

Description	Source / Repository	Persistent ID / URL
Lp(a) was measured using a latex enhanced immunoturbidimetric assay in 1996	Randox Laboratories	https://randox.com

Table S1: Stable isotope-labelled standards (SIS)

Protein	AQUA Ultimate peptide sequence	Concentration (nM)
Apo(a)-CR	LFLEPTQADIALLK[+8]	75
Apo(a)-KR	GTYSTTVTGR[+10]	60
ApoA1	VQPYLDDFQK[+8]	75
ApoA2	EQLTPLIK[+8]	56
ApoA4	LAPLAEDVR[+10]	37
ApoA4	LGEVNTYAGDLQK[+8]	52
ApoB	TEVIPPLIENR[+10]	19
ApoC1	EFGNTLEDK[+8]	37
ApoC2	TAAQNLYEK[+8]	37
ApoC3	GWVTDGFSSLK[+8]	149
ApoD	NILTSNNIDVK[+8]	37
ApoE	AATVGSLAGQPLQER[+10]	37
ApoH	ATVVYQGER[+10]	75
ApoL1	ALDNLAR[+10]	7
ApoM	FLLYNR[+10]	22

AQUA Ultimate peptides were obtained from Thermo Scientific. Arginine and lysine residues of the peptides are labelled with ^{13}C and ^{15}N atoms. Values in square brackets refer to the approximate mass shift in Dalton of a given SIS peptide compared to the unlabelled peptide. Apo, apolipoprotein; CR, constant region; KR, kringle IV-type 2 repeat.

Table S2: Baseline characteristics of the ASCOT trial participants, by allocation to statin treatment

	Treatment group (Mean (SD) or n (%))	Placebo group (Mean (SD) or n (%))	P-value*
No. of participants	20	20	
Age, years	63.3 (6.9)	64.0 (7.3)	0.732
Sex, n (%) female	4 (20%)	0 (0%)	0.106
Diabetes	1 (5%)	2 (10%)	1
Hypertension	20 (100%)	20 (100%)	1
Systolic blood pressure, mmHg	156.9 (15.1)	164.3 (17.1)	0.158
Diastolic blood pressure, mmHg	95.6 (7.29)	94.9 (9.4)	0.807
Body mass index, kg/m^2	28.6 (4.1)	28.3 (3.6)	0.811
Current smoker	5 (25%)	3 (15%)	0.695
Prior Statin	0 (0%)	0 (0%)	1
Prior CVD	2 (10%)	4 (20%)	0.661
Conventional Lipids			
LDL-C, mmol/L	3.31 (0.96)	3.36 (0.51)	0.858
HDL-C, mmol/L	1.26 (0.37)	1.44 (0.43)	0.156
Triglycerides	1.91 (1.66)	1.71 (0.80)	0.631

*P-values estimated using Student's t-test and Fisher's exact test for continuous and categorical variables respectively. SD, standard deviation; No., number; LDL-C, low-density lipoprotein cholesterol; HDL-C, high-density lipoprotein cholesterol; CVD, cardiovascular disease; kg/m2, kilograms per metre squared; mmHg, millimetres of mercury; mmol/L, millimoles per litre.

Table S3: Mean values of apolipoproteins and conventional lipids before and after statin therapy, and percentage change after statin therapy in 20 participants allocated to statin treatment in the ASCOT trial

	Off-statin (Baseline)		On-statin (1 year)		
	Mean	SD	Mean	SD	Percentage change (95% CI)
LP(a) related, nmol/L					
Apo(a) KR	646.80	782.26	657.20	777.83	1.6 (-1.1, 4.3)
Apo(a) CR	29.22	58.67	40.91	61.39	40.0 (20.1, 59.9)
LDL-related					
LDL-C, mmol/L	3.48	0.56	1.99	0.40	-43.2 (-46.5, -39.9)
ApoB, mg/L	647.1	103.11	401.3	87.02	-38.5 (-42.4, -34.5)
Triglyceride-related					
Triglycerides, mmol/L	1.91	1.66	1.22	0.56	-29.9 (-39.5, -20.2)
ApoC1, mg/L	1.27	0.51	0.69	0.35	-46.4 (-56.2, -36.6)
ApoC2, mg/L	15.04	6.69	10.31	3.75	-29.1 (-37.4, -20.8)
ApoC3, mg/L	126.9	45.95	81.09	25.45	-35.5 (-40.9, -30.2)
ApoE, mg/L	18.89	9.14	11.94	4.28	-33.4 (-41.8, -25.0)
HDL-related					
HDL-C, mmol/L	1.25	0.37	1.32	0.42	4.5 (-1.9, 10.8)
ApoA1, mg/L	862.45	144.05	866.6	190.58	-0.6 (-6.7, 5.5)
ApoA2, mg/L	126.87	25.73	117.91	31.81	-8.5 (-17.0, 0.0)
ApoA4, mg/L	41.21	12.45	37.5	13.97	-10.4 (-22.0,1.1)
ApoD, mg/L	25.46	6.72	21.83	6.25	-14.4 (-19.8, -8.9)
ApoH, mg/L	29.18	5.72	29.16	6.25	-0.3 (-6.0, 5.5)
ApoL1, mg/L	12.46	3.14	9.91	2.85	-21.1 (-25.0, -17.2)
ApoM, mg/L	18.27	3.63	16.55	4.83	-11.2 (-20.5, -2.0)

SD, standard deviation; CI, confidence interval; LDL-C, low-density lipoprotein cholesterol; HDL-C, high-density lipoprotein cholesterol; mg/L, milligrams per litre; mmol/L, millimoles per litre; nmol/L, nanomoles per litre.

Table S4: Number of cases and controls, cutoff points and median values for quintiles of apolipoproteins and conventional lipids in PROCARDIS

Protein	Controls / Cases	Quintiles of protein level I	II	III	IV	V	Overall
Lp(a),	N	195 / 151	195 / 132	195 / 151	195 / 228	195 / 279	975 / 941
mg/dL	Median	2.9 / 2.8	6.3 / 6.4	9.7 / 9.8	16.9 / 17.2	47.2 / 53.5	9.7 / 13.2
	Cutpoints*	< 4.5	4.5 - 7.9	7.9 - 12.2	12.2 - 27.8	> 27.8	
Apo(a) KR,	N	274 / 216	115 / 82	195 / 146	193 / 194	195 / 303	972 / 941
nmol/L	Median	104.0 / 104.0	125.0 / 127.5	203.0 / 199.5	423.0 / 418.5	977.0 / 1110.0	203.0 / 302.0
	Cutpoints*	< 104.0	104.0 - 149.4	149.4 - 272.0	272.0 - 652.6	> 652.6	
Apo(a) CR,	N	25 / 35	24 / 40	24 / 27	24 / 44	25 / 78	122 / 224
nmol/L	Median	733 / 71.3	87.7 / 89.4	112.5 / 111.0	136.0 / 134.0	185.0 / 181.5	112.5 / 126.0
	Cutpoints*	< 80.1	80.1 - 102.4	102.4 - 121.6	121.6 - 150.8	> 150.8	
LDL-C,	N	198 / 154	192 / 172	195 / 149	199 / 176	191 / 290	975 / 941
mmol/L	Median	2.4 / 2.3	2.9 / 2.9	3.3 / 3.3	3.7 / 3.7	4.3 / 4.5	3.3 / 3.5
	Cutpoints*	< 2.7	2.7 - 3.1	3.1 - 3.5	3.5 - 4.0	> 4.0	
ApoB,	N	195 / 87	195 / 139	195 / 159	195 / 189	195 / 367	975 / 941
mg/L	Median	522.0 / 517.9	648.0 / 646.6	745.0 / 749.1	858.0 / 862.6	1060.0 / 1130.0	744.0 / 857.0
	Cutpoints*	< 586.8	586.8 - 697.6	697.6 - 798.0	798.0 - 930.2	> 930.2	
Triglycerides,	N	197 / 45	193 / 96	195 / 150	195 / 231	195 / 419	975 / 941
mmol/L	Median	0.8 / 0.8	1.0 / 1.1	1.4 / 1.4	1.9 / 1.9	3.0 / 3.5	1.4 / 2.1
	Cutpoints*	< 0.9	0.9 - 1.2	1.2 - 1.6	1.6 - 2.3	> 2.3	
ApoC1,	N	195 / 155	210 / 142	186 / 167	189 / 153	195 / 324	975 / 941
mg/L	Median	7.5 / 7.6	9.8 / 9.7	11.3 / 11.4	12.9 / 13.2	15.7 / 17.0	11.1 / 12.3
	Cutpoints*	< 0.9	0.9 - 1.2	1.2 - 1.6	1.6 - 2.3	> 2.3	
ApoC2,	N	195 / 63	196 / 114	197 / 138	192 / 218	195 / 408	975 / 941
mg/L	Median	6.0 / 6.1	8.0 / 8.1	9.9 / 10.2	12.4 / 12.7	17.3 / 18.5	9.9 / 13.3
	Cutpoints*	< 7.1	7.1 - 9.0	9.0 - 11.0	11.0 - 14.2	> 14.2	
ApoC3,	N	196 / 93	195 / 81	196 / 161	202 / 180	186 / 426	975 / 941
mg/L	Median	50.2 / 48.6	65.9 / 65.0	81.0 / 81.3	101.5 / 100.2	134.5 / 153.2	80.7 / 106.9
	Cutpoints*	< 58.3	58.3 - 71.9	71.9 - 90.3	90.3 - 112.0	> 112.0	
ApoE,	N	203 / 92	195 / 133	188 / 155	194 / 201	195 / 360	975 / 941
mg/L	Median	17.7 / 17.7	21.8 / 22.0	25.5 / 25.5	29.9 / 30.0	39.2 / 40.2	25.4 / 29.9
	Cutpoints*	< 19.9	19.9 - 23.5	23.5 - 27.3	27.3 - 33.2	> 33.2	
HDL-C,	N	205 / 396	192 / 247	189 / 142	195 / 91	194 / 65	975 / 941
mmol/L	Median	0.9 / 0.9	1.2 / 1.1	1.3 / 1.3	1.5 / 1.5	1.9 / 1.9	1.3 / 1.1
	Cutpoints*	< 1.0	1.0 - 1.2	1.2 - 1.4	1.4 - 1.7	> 1.7	

ApoA1,	N	196 / 324	196 / 213	193 / 166	195 / 147	195 / 91	975 / 941
mg/L	Median	671.0 / 654.5	775.0 / 771.0	862.0 / 857.0	959.0 / 961.0	1090.0 / 1090.0	862.0 / 789.0
	Cutpoints*	< 730.0	730.0 - 820.0	820.0 - 908.4	908.4 - 1012.0	> 1012.0	
ApoA2,	N	196 / 273	196 / 206	197 / 159	191 / 138	195 / 165	975 / 941
mg/L	Median	30.1 / 29.5	36.3 / 36.8	41.8 / 42.1	47.5 / 47.1	57.5 / 57.7	41.8 / 39.3
	Cutpoints*	< 33.6	33.6 - 39.6	39.6 - 44.5	44.5 - 51.6	> 51.6	
ApoA4,	N	196 / 278	197 / 174	194 / 138	195 / 175	193 / 176	975 / 941
mg/L	Median	42.4 / 40.5	51.9 / 51.2	59.0 / 58.0	67.7 / 67.1	84.6 / 85.3	58.9 / 55.8
	Cutpoints*	< 47.2	47.2 - 54.8	54.8 - 62.3	62.3 - 74.2	> 74.2	
ApoD,	N	196 / 348	197 / 173	195 / 173	195 / 109	192 / 138	975 / 941
mg/L	Median	30.5 / 28.7	36.7 / 36.2	41.4 / 40.9	46.9 / 46.5	55.9 / 57.0	41.3 / 37.3
	Cutpoints*	< 33.8	33.8 - 38.8	38.8 - 44.1	44.1 - 50.4	> 50.4	
ApoH,	N	196 / 153	212 / 171	184 / 149	188 / 189	195 / 279	975 / 941
mg/L	Median	86.4 / 85.6	104.0 / 104.0	115.0 / 115.0	126.0 / 126.0	143.0 / 145.0	114.0 / 119.0
	Cutpoints*	< 97.4	97.4 - 110.0	110.0 - 119.0	119.0 - 132.2	> 132.2	
ApoL1,	N	204 / 114	186 / 149	197 / 171	196 / 202	192 / 305	975 / 941
mg/L	Median	10.6 / 10.5	13.0 / 13.2	15.4 / 15.3	17.9 / 17.7	22.6 / 22.8	15.4 / 16.8
	Cutpoints*	< 11.9	11.9 - 14.3	14.3 - 16.4	16.4 - 19.6	> 19.6	
ApoM,	N	200 / 381	192 / 161	198 / 155	195 / 145	190 / 99	975 / 941
mg/L	Median	15.4 / 14.6	18.5 / 18.3	20.7 / 20.7	23.3 / 23.2	28.1 / 27.7	20.7 / 18.5
	Cutpoints*	< 17.1	17.1 - 19.4	19.4 - 21.8	21.8 - 25.1	> 25.1	

SD, standard deviation; CI, confidence interval; LDL-C, low-density lipoprotein cholesterol; HDL-C, high-density lipoprotein cholesterol; mg/L, milligrams per litre; mmol/L, millimoles per litre; nmol/L, nanomoles per litre.

Table S5: Comparison of risk of CHD for top versus bottom quintiles of apolipoproteins and conventional lipids adjusting for CHD risk factors and conventional lipids vs adjusting for CHD risk factors and conventional lipids and BMI

	**Adjusted for CHD risk factors + lipids OR (95% CI)**	**Adjusted for CHD risk factors + lipids + BMI OR (95% CI)**
Lp(a)-related		
Lp(a)	2.07 (1.45, 2.95)	2.22 (1.54, 3.19)
Apo(a) kringle-repeat	2.84 (2.04, 3.95)	3.08 (2.19, 4.32)
Apo(a) constant-region	2.34 (0.82, 6.70)	2.14 (0.75, 6.07)
LDL-related*		
LDL-C	1.62 (1.12, 2.35)	1.58 (1.08, 2.31)
ApoB	1.98 (1.19, 3.32)	1.87 (1.11, 3.16)
Triglyceride-related*		
Triglycerides	3.76 (2.35, 6.00)	3.67 (2.27, 5.93)
ApoC1	2.38 (1.63, 3.46)	2.53 (1.72, 3.71)
ApoC2	1.82 (1.17, 2.81)	1.79 (1.15, 2.79)
ApoC3	2.95 (1.85, 4.71)	3.16 (1.97, 5.09)
ApoE	2.35 (1.54, 3.58)	2.13 (1.39, 3.28)
HDL-related		
HDL-C	0.32 (0.20, 0.49)	0.34 (0.21, 0.54)
ApoA1	0.63 (0.39, 1.03)	0.59 (0.36, 0.97)
ApoA2	0.78 (0.53, 1.13)	0.79 (0.54, 1.17)
ApoA4	0.45 (0.31, 0.65)	0.43 (0.30, 0.62)
ApoD	0.77 (0.52, 1.15)	0.78 (0.52, 1.17)
ApoH	0.90 (0.63, 1.28)	0.88 (0.61, 1.27)
ApoL1*	1.77 (1.21, 2.59)	1.75 (1.18, 2.58)
ApoM	0.29 (0.19, 0.44)	0.29 (0.19, 0.45)

Variables with an asterisk (*) are corrected for statin use. All estimates adjusted for age, sex, country, smoking, diabetes, hypertension, hours since last meal, LDL-C, HDL-C, and triglycerides. OR, odds ratio; CI; confidence interval; LDL-C, low-density lipoprotein cholesterol; HDL-C, high-density lipoprotein cholesterol; Lp(a), lipoprotein(a); Apo, apolipoprotein; BMI, body mass index

Table S6: Comparison of risk of CHD for top versus bottom quintiles of apolipoproteins and conventional lipids for all participants in PROCARDIS and a subset without diabetes mellitus

	**All participants (n = 1,916) OR (95% CI)**	**Participants without diabetes (n = 1,748) OR (95% CI)**
Lp(a)-related		
Lp(a)	1.90 (1.37, 2.64)	1.94 (1.38, 2.72)
Apo(a) kringle-repeat	2.11 (1.57, 2.83)	2.14 (1.58, 2.90)
Apo(a) constant-region	1.78 (0.70, 4.53)	1.78 (0.70, 4.53)
LDL-related*		
LDL-C	2.13 (1.53, 2.95)	1.90 (1.37, 2.64)
ApoB	3.74 (2.62, 5.35)	3.47 (2.43, 4.97)
Triglyceride-related*		
Triglycerides	7.99 (5.34, 11.96)	8.06 (5.32, 12.20)
ApoC1	2.97 (2.12, 4.17)	2.91 (2.07, 4.09)
ApoC2	4.71 (3.26, 6.79)	4.74 (3.27, 6.86)
ApoC3	4.41 (3.09, 6.30)	4.22 (2.95, 6.02)
ApoE	4.02 (2.81, 5.77)	3.94 (2.75, 5.66)
HDL-related		
HDL-C	0.17 (0.12, 0.25)	0.16 (0.11, 0.24)
ApoA1	0.30 (0.21, 0.44)	0.29 (0.19, 0.43)
ApoA2	0.69 (0.50, 0.95)	0.66 (0.48, 0.92)
ApoA4	0.43 (0.31, 0.60)	0.41 (0.29, 0.58)
ApoD	0.42 (0.30, 0.59)	0.40 (0.29, 0.57)
ApoH	1.24 (0.90, 1.72)	1.22 (0.88, 1.71)
ApoL1*	2.92 (2.05, 4.16)	2.55 (1.79, 3.64)
ApoM	0.28 (0.19, 0.40)	0.27 (0.18, 0.38)

Variables with an asterisk (*) are corrected for statin use. All estimates adjusted for age, sex, country, smoking, diabetes, hypertension, and hours since last meal. OR, odds ratio; CI; confidence interval; LDL-C, low-density lipoprotein cholesterol; HDL-C, high-density lipoprotein cholesterol; Lp(a), lipoprotein(a); Apo, apolipoprotein.

Table S7: Baseline characteristics of the Bruneck study participants

Characteristics	Mean (SD) or n (%)
No. of participants	688
Age, years	66.0 (10.2)
Sex, n (%) female	357 (52%)
Country	
Italy	688 (100%)
Diabetes	74 (11%)
Hypertension	206 (30%)
Systolic blood pressure, mmHg	139.7 (18.7)
Diastolic blood pressure, mmHg	83.9 (8.3)
Body mass index, kg/m^2	26.7 (4.0)
Current smoker	112 (16%)
Statin use	66 (10%)
Prior CVD	44 (6%)
Conventional lipids	
LDL-C, mmol/L	3.85 (0.43)
HDL-C, mmol/L	1.49 (0.39)
Triglycerides, mmol/L	1.55 (0.86)

SD, standard deviation; No., number; LDL-C, low-density lipoprotein cholesterol; HDL-C, high-density lipoprotein cholesterol; CVD, cardiovascular disease; kg/m2, kilograms per metre squared; mmHg, millimetres of mercury; mmol/L, millimoles per litre

Figure S1: Mean plasma concentrations of apolipoproteins and conventional lipids at baseline and one year in ASCOT, by statin allocation

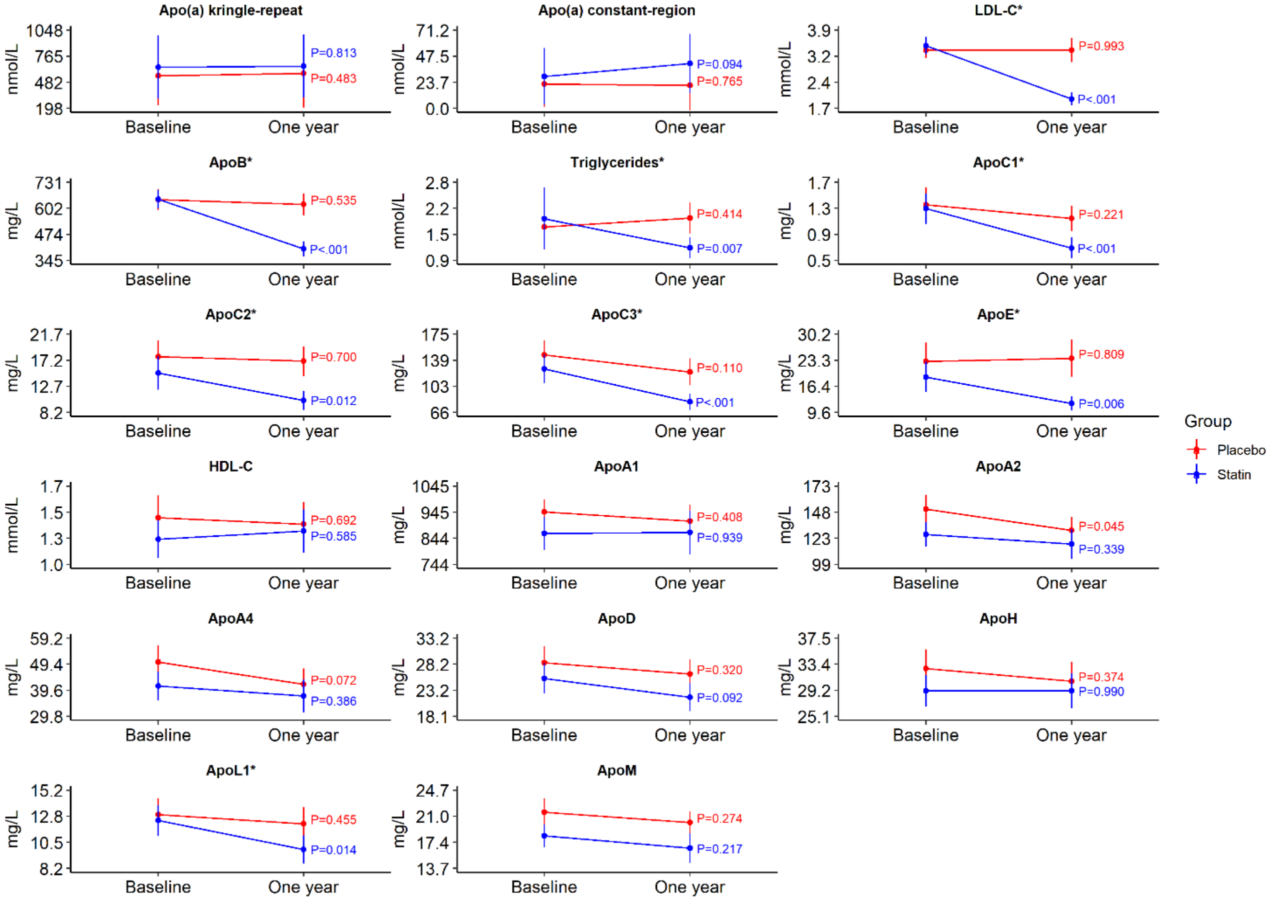

*Meets criteria for statin correction

P-values estimated using paired sample t-tests. P, P-value; LDL-C, low-density lipoprotein cholesterol; HDL, high-density lipoprotein cholesterol; Lp(a) , lipoprotein(a); Apo, apolipoprotein; mg/L, milligrams per litre; mmol/L, millimoles per litre; nmol/L, nanomoles per litre.

Figure S2: Comparison of CHD risk for top versus bottom quintiles of apolipoproteins and conventional lipids, before and after correction for statin use in PROCARDIS

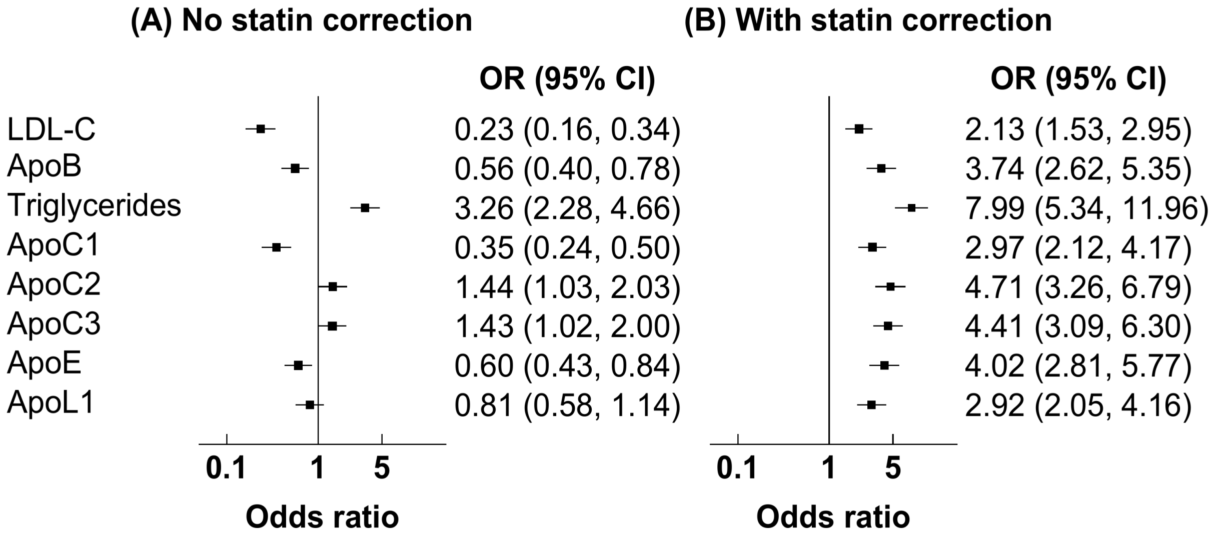

All estimates adjusted for age, sex, country, smoking, diabetes, hypertension, and hours since last meal. OR, odds ratio; CI; confidence interval; LDL-C, low-density lipoprotein cholesterol.

Figure S3: Comparison of risk of CHD for a one standard deviation difference in apolipoproteins and conventional lipids in PROCARDIS and the Bruneck study

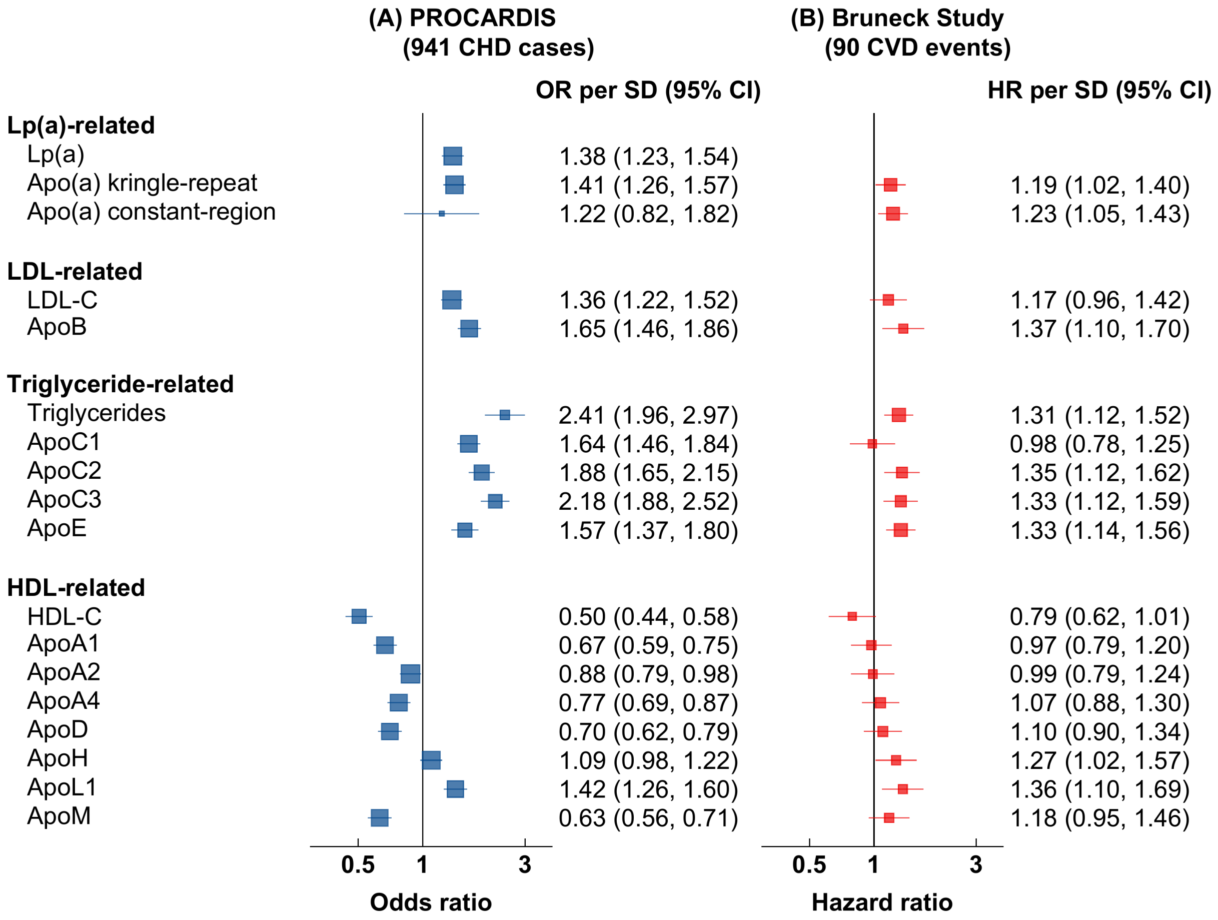

Complete data were available for all measurements in 941 cases and 975 controls in PROCARDIS, with the exception of Apo(a) KR (941 cases/972 controls) and Apo(a) CR (224 cases/122 controls). PROCARDIS estimates are adjusted for age, sex, country, smoking, diabetes, hypertension, and hours since last meal. Participant level values for LDL-C, ApoB, triglycerides, ApoC1, ApoC2, ApoC3, ApoE, and ApoL1 are corrected for statin use. Complete data were available in the Bruneck study, with the exception of Apo(a) CR: 19/90 (21.1%) participants with CVD events and 89/598 (14.9%) participants without CVD events had Apo(a) CR above limit of detection. Bruneck estimates are adjusted for age, sex, smoking, diabetes, hypertension, and statin use. Boxes represent effect sizes with their size inversely proportional to variance. Abbreviations: CHD, coronary heart disease; CVD, cardiovascular disease; OR, odds ratio; HR, hazard ratio; CI; confidence interval; LDL-C, low-density lipoprotein cholesterol; HDL-C, high-density lipoprotein cholesterol; Lp(a), lipoprotein(a); Apo, apolipoprotein.
